# Mapping the Evolution of China’s Traditional Chinese Medicine Education Policies: Insights From a BERTopic-Based Descriptive Study

**DOI:** 10.2196/72660

**Published:** 2025-09-25

**Authors:** Tao Yang, Fan Yang, Yong Li

**Affiliations:** 1 School of Basic Medical Sciences, Chengdu University of Traditional Chinese Medicine, Chengdu, China; 2 School of Management, Chengdu University of Traditional Chinese Medicine, Sichuan Province, Chengdu, 611137, China, 86 15828528952; 3 School of Teacher Education, East China Normal University, Shanghai, China

**Keywords:** traditional Chinese medicine education policy, BERTopic, policy evolution, descriptive study, topic analysis

## Abstract

**Background:**

Traditional Chinese medicine (TCM) education in China has evolved significantly, shaped by both national policy and social needs. Despite this, the academic community has yet to fully explore the long-term trends and core issues in TCM education policies. As the global interest in TCM continues to grow, understanding these trends becomes crucial for guiding future policy and educational reforms. This study used cutting-edge deep learning techniques to fill this gap, offering a novel, data-driven perspective on the evolution of TCM education policies.

**Objective:**

This study aimed to systematically analyze the research topics and evolutionary trends in TCM education policies in China using a deep learning–based topic modeling approach, providing valuable insights to guide future policy development and educational practices.

**Methods:**

TCM policy–related documents were collected from major sources, including the Ministry of Education, the National Administration of Traditional Chinese Medicine, PKU Lawinfo, and archives of TCM colleges. The text was preprocessed and analyzed using the BERTopic model, a state-of-the-art tool for topic modeling, to extract key themes and examine the policy development trajectory.

**Results:**

The analysis revealed 27 core topics in TCM education policies, including medical education, curriculum reform, rural health care, internationalization, and the integration of TCM with modern education systems. These topics were clustered into 5 stages of policy evolution: marginalization, standardization, specialization, systematization, and restandardization. These stages reflect the ongoing balancing act between modernizing TCM education and preserving its traditional values, while adapting to national political, social, and economic strategies.

**Conclusions:**

This study offers groundbreaking insights into the dynamic and multifaceted evolution of TCM education policies in China. By leveraging the BERTopic model, it provides a comprehensive framework for understanding the forces shaping TCM education and offers actionable recommendations for future policy making. The findings are essential for educators, policymakers, and researchers aiming to refine and innovate TCM education in an increasingly globalized world.

## Introduction

Traditional Chinese medicine (TCM) is an important part of China’s 5000-year-old culture. Its education policy not only concerns the inheritance and development of TCM but also has a profound impact on the global health system [1]. In the modernization process of China, the evolution of TCM education policies reflects the country’s emphasis on and adjustment of traditional medical education, as well as the complex interaction between social demands, political environment, and cultural confidence. With the enhancement of China’s comprehensive national power and its rising international status, TCM education has gradually become an important part of global health governance. The direction and effectiveness of its policies directly affect the influence and adaptability of TCM globally [1]. Against this backdrop, in-depth research and analysis of the evolution and development path of TCM education policies are of great academic and practical significance. By tracing the evolution of TCM education policies, we can reveal the driving forces and logical relationships behind the policies, providing a solid basis for decision makers. Moreover, analyzing the evolutionary trends of TCM education policies can also help educational institutions and researchers better understand the future direction of TCM education, thereby promoting the reform and innovation of TCM education to meet the diverse needs of modern society and international challenges.

Text mining technology, as an advanced data analysis method, can extract potential topics and patterns from a large number of policy texts, providing a new perspective for understanding the evolution of TCM education policies. Through systematic analysis of policy texts, we can identify the changing trends, weight distribution, and logical structure of policy topics, thereby revealing the focus and orientation of TCM education policies in different historical stages. This analysis not only provides empirical support for the theoretical research of TCM education but also offers scientific references for policymakers when formulating future TCM education policies [2]. This study uses Bert and dynamic topic modeling (DTM) and other text mining methods to conduct topic mining [3,4], identification, and analysis of TCM education policy texts to study the development direction and evolutionary path of TCM education policies, providing guidance and references for scientific research and scientific management practices in relevant fields.

## Methods

### Data Sources

This study obtained more than 200 national-level policy texts related to TCM education from 1902 to 2024 from the official websites of the Central People’s Government of the People’s Republic of China, the Ministry of Education, the National Health Commission, and the National Administration of Traditional Chinese Medicine; China National Knowledge Infrastructure (including yearbooks, mainly the China TCM Yearbook); publicly published books, journals, and newspapers; and internal material compilations and archive files of relevant departments, supplemented by important policies mentioned in literature studies. These data cover key stages and important nodes of TCM education policies, ensuring the comprehensiveness and systematic nature of the study.

### Inclusion Criteria

Only national-level policy documents directly related to TCM education were included. This means policies that explicitly addressed TCM educational objectives, curriculum design, teaching methods, teacher qualifications, student enrollment, or educational resource allocation in the context of TCM education were selected. Policies that were tangentially related, such as general health policies with only a passing mention of TCM without specific educational implications, were excluded.

### Exclusion Criteria

Documents that were not official policies, such as opinion pieces, academic commentaries, and nongovernmental reports on TCM education, were excluded. Additionally, policies that were duplicates, drafts, or had been superseded by subsequent policies were not included in the final dataset.

The data collection focuses on national-level policy documents, combined with TCM education reform plans and guiding opinions issued by the Ministry of Education and health departments, as well as relevant historical records and academic research reports, to build a complete policy evolution database.

### Overall Research Framework

The overall research framework of this paper is divided into data collection, model improvement, data cleaning, BERTopic data processing, result output, and result interpretation. BERTopic is a topic modeling technique based on BERT word vectors [5], which creates dense clusters using Transformers and class-based Term Frequency–Inverse Document Frequency (c-TF-IDF) while retaining important words in topic descriptions. The topic modeling analysis using this model can be carried out in the following 5 steps: embeddings, dimensionality reduction, clustering, tokenizer, and weighting scheme [6]. The model has modular features, and processing methods can be chosen autonomously at each step according to research needs [7].

Compared with traditional topic models such as Latent Dirichlet Allocation (LDA), BERTopic exhibits distinct advantages in analyzing TCM policies. Traditional models rely on statistical co-occurrence of words, which often fails to capture semantic relationships effectively. For instance, LDA assumes that topics are generated from a Dirichlet distribution over words, struggling to differentiate between polysemous terms [8]. In contrast, BERTopic leverages pretrained BERT embeddings, enabling it to understand the context of words, thus better handling TCM-specific terminologies that are often ambiguous and context-dependent. BERTopic, by integrating c-TF-IDF, emphasizes the importance of words within topics based on semantic similarity, leading to more interpretable and accurate topic representations in the TCM policy domain [5].

When compared with existing TCM policy research using methods such as LDA-based analysis, BERTopic demonstrates significant superiority in semantic understanding. Traditional LDA-based studies often produce topics with overlapping meanings and struggle to distinguish between terms with similar statistical distributions but different semantic interpretations [9]. For example, in TCM policies, terms such as “herbal medicine” may have multiple connotations, such as medicinal plant resources, prescription formulations, or quality control standards. BERTopic, powered by its deep bidirectional Transformer architecture, can analyze the surrounding text to disambiguate these polysemous terms accurately [10]. Moreover, TCM has a rich set of domain-specific jargon, and BERTopic’s pretraining on extensive language corpora allows it to recognize and leverage these specialized terminologies more effectively, providing a more nuanced and comprehensive understanding of TCM policy documents [11].

### Data Cleaning and BERTopic Model Improvement Methods

After obtaining the TCM education policy-related texts, since the BERTopic model itself has a limit on text length, it will truncate texts exceeding 512 characters. However, most policy texts have more than 1000 characters. Therefore, this study improved the BERTopic model by extracting BERT features for every 512 characters of long texts and then calculating the mean value as the text feature.

In addition, conventional data cleaning of policy texts is also required. First, the text content of Word and PDF policy documents is extracted, and obvious recognition errors and redundant invalid data are removed or replaced. Then, using the Jieba library and the HIT stop word list, common Chinese stop words are removed. Furthermore, a specialized word dictionary for the TCM policy field is used to segment the text, obtaining preprocessed text data.

### Data Processing Methods

#### Text Vectorization

The first step in processing the text input into the model is text vectorization, that is, embedding. Before conducting topic modeling and clustering, it is necessary to convert text data into numerical vector representations, which is the basis of topic modeling [12]. In the BERTopic constructed in this study, the preprocessed documents are converted into dense vector representations based on the Sentence-BERT framework. By adding pooling operations in the output of BERT, fixed-size sentence embeddings are obtained. This study uses the paraphrase-multilingual-MiniLM-L12-V2 sentence vector model to represent the preprocessed text embeddings. This model supports more than 50 languages, including Chinese, and is very effective for most use cases. It not only greatly improves training speed but also maintains superior performance.

#### Dimensionality Reduction

After text vectorization, dimensionality reduction is required, that is, mapping high-dimensional data to low-dimensional space. The purpose of dimensionality reduction is to reduce the dimension of the embedding vectors, thereby simplifying computation and storage and enhancing clustering effects [13]. Common dimensionality reduction techniques include Uniform Manifold Approximation and Projection (UMAP), Principal Component Analysis, and t-Distributed Stochastic Neighbor Embedding, with UMAP being the default choice for BERTopic. This method effectively reduces dimensions while retaining the local and global structure of the data, ensuring the accuracy of clustering semantically similar documents. Compared with other dimensionality reduction algorithms, UMAP is better at preserving the characteristics of the original data in low-dimensional space, making it the preferred method for text clustering dimensionality reduction.

#### Clustering

After text dimensionality reduction, the next step is to use clustering algorithms to assign embedding vectors to different topics. In this study, the HDBSCAN (Hierarchical Density-Based Spatial Clustering of Applications with Noise) algorithm is used for topic clustering of the reduced dimension document embeddings [14]. HDBSCAN extends DBSCAN (Density-Based Spatial Clustering of Applications with Noise) into a hierarchical clustering algorithm, which can automatically select the optimal clusters and treat noise as outliers, thereby avoiding the incorrect allocation of unrelated documents. This algorithm can generate dynamic topic representations, allowing the same topic to be expressed in different forms at different times, providing flexibility for studying the temporal changes of policy topics [15].

#### Topic Word Extraction

After topic clustering is completed, the next step is to extract and represent topic words using the Bag-of-Words model. This study used the c-TF-IDF method, which combines all documents in each cluster into a long document and calculates the frequency of each word [5]. To improve the coherence and diversity of topics, maximal marginal relevance is further applied to optimize the selection of topic words, reducing the repetition of synonyms and ensuring the diversity and accuracy of topic representation.

#### Dynamic Topic Modeling

DTM takes into account the changes in topics over time and can better capture the evolution of policies than static topic modeling [16]. BERTopic combines temporal factors by calculating topic representations at each time point to achieve DTM. First, the model is fitted globally and then the topic representations at each time point are fine-tuned. This allows for more precise analysis of topic evolution through global and evolutionary adjustments, providing an effective tool for understanding changes in policy topics over different periods.

## Results

### TCM Education Policy Topic Extraction Results

Using the BERTopic model for topic modeling of policy abstracts, this study adjusted the model parameters based on multiple experimental results. Under the set parameter conditions, a total of 27 main research topics in TCM education policies were identified (from topic 0 to topic 26), covering 183 policy texts in the dataset. Another 27 policy texts were classified as noise data (assigned to the −1 category). The noise data, aside from a few outlier policy texts, mainly consisted of research topics that did not reach the set parameter cluster size. These were mostly secondary or minor topics under the main themes, and their impact on the main research topics of TCM education policies was minimal. The representative keywords generated by the model for each topic are shown in Table 1.

**Table 1.** Topic identification results and related document counts based on BERTopic.

Topic number	Document count	Percentage	Keywords
Topic 0	20	10.9	“Training,” “Medical Education,” “Clinical,” “Method,” “Major,” “Outpatient,” “Technology,” “Principle,” “Content,” and “Trainee”
Topic 1	14	7.7	“Class Hours,” “Lecture,” “‘Chinese Medicine,” “Labor,” “Teaching Plan,” “Student,” “Basic,” “Assessment,” “Teaching,” and “Medic”
Topic 2	10	5.5	“Project,” “Base,” “Education,” “Credit,” “Management,” “Certificate,” “Committee,” “Method,” “Sponsor,” and “Office”
Topic 3	10	5.5	“Talent,” “Education,” “Service,” “Field,” “Construction,” “Society,” “Hospital,” “Teaching,” “Development,” and “Clinical”
Topic 4	10	5.5	“Successor,” “Mentor,” “Acupuncture,” “Exam,” “Expert,” “Experience,” “International,” “Graduation,” “Academic,” and “Work”
Topic 5	9	5.0	“Education,” “School,” “Development,” “Teacher,” “Reform,” “Government,” “Society,” “Education,” “Vocational,” and “System”
Topic 6	9	5.0	“Rural,” “Village,” “Doctor,” “Team,” “Project,” “Work,” “Personnel,” “Rural Health,” “Plan,” and “Training”
Topic 7	9	5.0	“Graduate,” “Education,” “Vocation,” “Academic,” “University,” “Quality,” “Development,” “Reform,” and “Tutor”
Topic 8	9	5.0	“Reform,” “Model,” “Teaching,” “Training,” “Education,” “Medicine,” “Five-Year Program,” “Standardization,” “Construction,” and “General Practice“
Topic 9	9	5.0	“Graduate,” “Assessment,” “Degree,” “Bachelor’s Degree,” “University,” “Unit,” “Work,” “Quality,” and “Discipline”
Topic 10	9	5.0	“Medical Education,” “Medicine,” “Major,” “Ethnic Minority,” “Ministry of Education,” “Construction,” “Medical College,” “Student,” and “Teaching”
Topic 11	7	3.8	“Meeting,” ”Work,” “Enterprise,” “Development,” “Problem,” “Personnel,” “National,” “Reform,” “Western Medicine,” and “Comrade”
Topic 12	6	3.3	“Graduate,” “Education,” “Development,” “Student,” “School,” “Student,” “Vocation,” “Education,” “Guidance,” and “Society“
Topic 13	5	2.7	“Teaching,” “Physical Education,” “Ideology,” “Student,” “Part-time,” “Half-work,” “Medical Skill,” “Physical Education Class,” “Work,” and “Patient”
Topic 14	5	2.7	“Degree,” “Professional Degree,” “Class Hours,” “Experiment,” “Chinese Medicine,” “Medicine,” “Master’s Degree,” “Set,” “Clinical,” and “Assessment”
Topic 15	5	2.7	“Textbook,” “Question Setting,” “Question,” “Group,” “Question Bank,” “Work,” “Course,” “Database Construction,” “Question Bank,” and “Planning”
Topic 16	5	2.7	“School,” “Teaching,” “Regulation,” “School,” “Mathematics,” “History,” “Physics,” “Graduation,” “Geography,” and “Student”
Topic 17	4	2.2	“Research,” “Construction,” “Chinese Medicine,” “Service,” “Medicine,” “Development,” “AIDS,” “Advantage,” “Medical,” and “Key”
Topic 18	4	2.2	“University,” “College,” “Experiment,” “Chemistry,” “Department,” “President,” “Subject,” “Lecture Notes,” “Dean,” and “Enrollment”
Topic 19	4	2.2	“Major,” “Teaching,” “Setting,” “Basic,” “Hospital,” “Method,” “Full-time Teacher,” “Course,” “Pathogenesis,” and “Clinical”
Topic 20	4	2.2	“Service,” “Medical Institution,” “Chinese Medicinal Materials,” “Development,” “Construction,” “National,” “Medical,” “System,” “Research,” and “Institution”
Topic 21	3	1.6	“Assessment,” “Correspondence Education,” “Institution,” “Indicator System,” “Education,” “Report,” “Level,” “Work,” “Aspect,” and “Experience”
Topic 22	3	1.6	“International Student,” “Exam,” “Institution,” “Center,” “Education Commission,” “Graduation Certificate,” “Regulation,” “Time,” “Academic Certificate,” and “Question Setting”
Topic 23	3	1.6	“Chairman Mao,” “Team,” “Autonomous Region,” “New Medicine,” “Medical Science,” “Policy,” “Medicine,” “Work,” “Lack of Successors,” and “Important Instruction”
Topic 24	3	1.6	“Credit,” “Committee,” “Education,” “Academic Conference,” “Score,” “Chairman,” “Project,” “Secretary-General,” “Assessment,” and “Method”
Topic 25	2	1.1	“Talent,” “Construction,” “Service,” “Medicine,” “Development,” “Standardization,” “Chinese Medicine,” “Ethnic,” “Culture,” and “Talent Team”
Topic 26	2	1.1	“Professional Degree,” “Master,” “Standardization,” “Graduate,” “Base,” “Family Planning Commission,” “Training,” “Office,” “Collaboration,” and “Medicine and Education”

**Figure 1.**
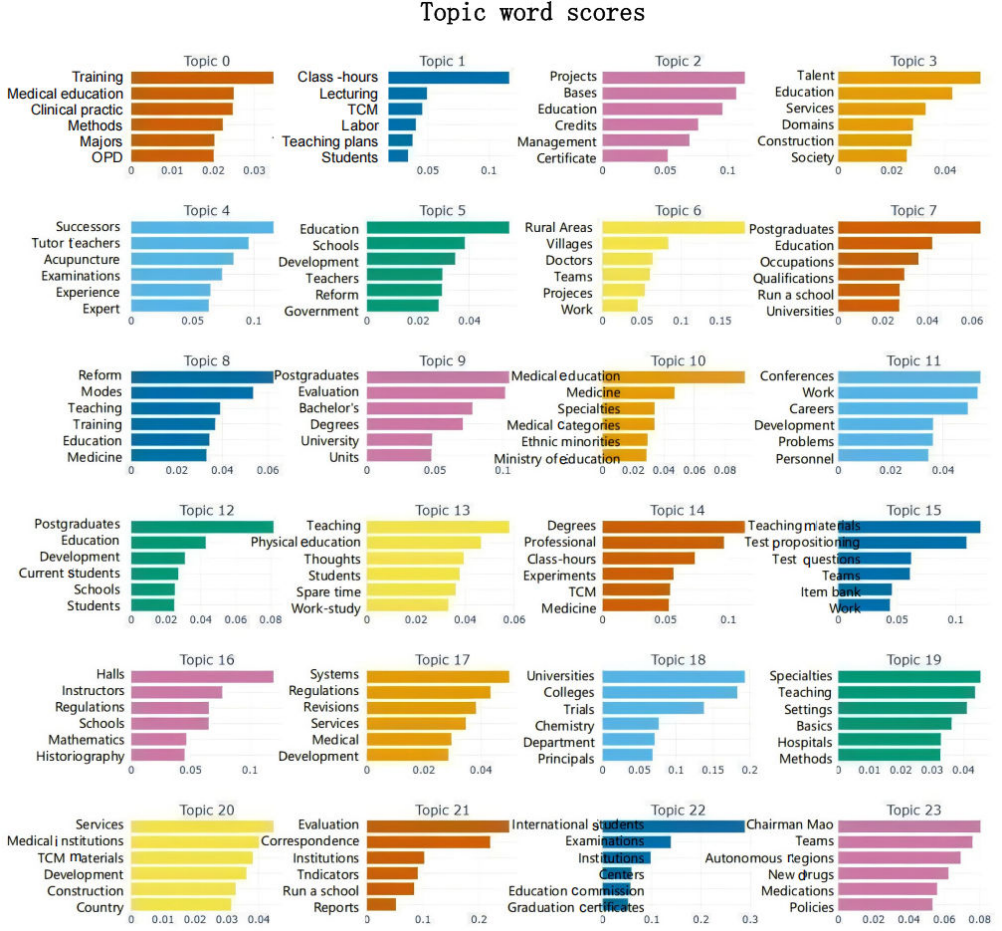
Visualization of traditional Chinese medicine education policy text topics. OPD: outpatient department; TCM: traditional Chinese medicine.

According to the results shown in Figure 1, the BERTopic model successfully extracted the main topics from the TCM education policy literature and effectively expressed these topics through feature words and weights. The following are specific descriptions of several topics in the figure: topic 3, with feature words such as “talent,” “education,” “service,” and “construction,” indicates that this topic focuses on the cultivation of TCM talents and the improvement of educational quality, showing the policy’s emphasis on enhancing the capabilities of TCM talents. Topic 6, with feature words including “rural,” “village,” “doctor,” and “team,” indicates that this topic is related to rural medical services and grassroots hospital construction, reflecting the efforts of TCM policies in promoting grassroots medical services. Topic 10, with feature words such as “medical education,” “Ministry of Education,” “medical major,” and “professional,” indicates that this topic mainly involves educational planning and professional construction related to the Ministry of Education, reflecting the focus of TCM education policies on overall planning and disciplinary development. Topic 14, with feature words such as “class hours,” “experiment,” and “degree,” indicates that this topic is mainly related to the degree settings and graduate education in TCM education, reflecting the policy inclination toward specialized cultivation in higher education. Topic 18, with feature words such as “university,” “college,” “department,” and “president,” focuses on the academic development and overall educational quality of TCM higher education institutions, showing the role of policies in promoting the systematic development of TCM education. Topic 21, with feature words such as “assessment,” “institutions,” “indicator system,” and “education,” indicates that this topic is related to the assessment policies of TCM education, focusing on the standardization of TCM education. Through these feature words and their weight distributions, it can be seen that the BERTopic model has effectively captured the differences between various topics and can accurately express the core content of each topic. These topics reflect the multiple dimensions of concern in TCM education policies, including educational planning, talent cultivation, grassroots medical services, systematization, specialization, and standardization. This provides a clear direction and basis for subsequent policy analysis and improvement.

**Figure 2.**
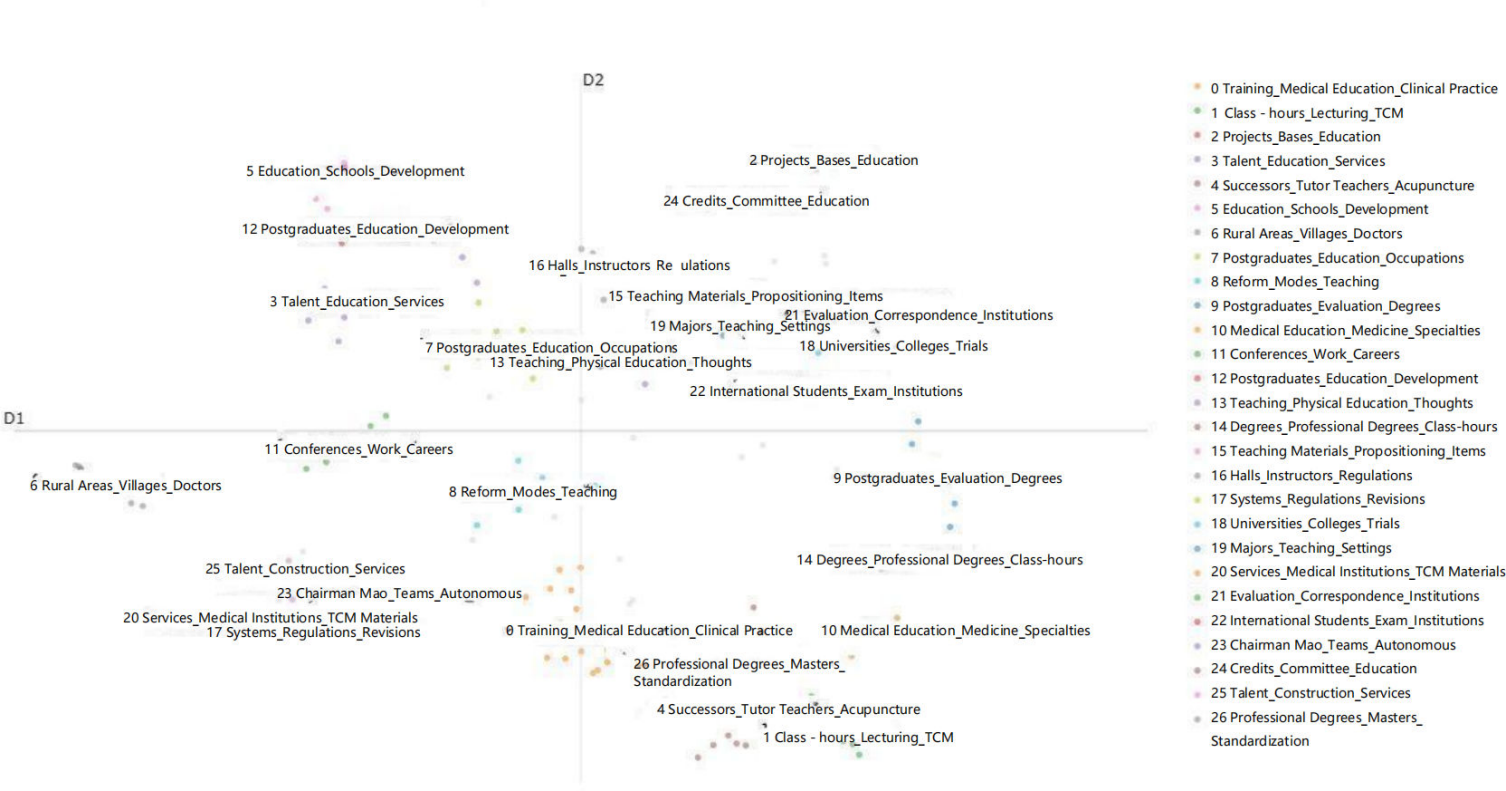
Thematic—term-clustering diagram. TCM: traditional Chinese medicine.

According to the 2D visualization distribution results of the topic-related documents shown in Figure 2, the clustering of different topics in the 2D space can be observed. The documents in the figure are roughly distributed in 4 quadrants, with each quadrant concentrating a category of topic documents, showing a clear trend of classification concentration. The first quadrant mainly focuses on topics related to educational curriculum reform and development. These documents involve the optimization of the educational system, curriculum reform, and the improvement of graduate education, reflecting the attention of TCM education policies on the development of the educational system. The second quadrant gathers topics related to academic research and teaching quality. These documents mainly focus on improving teaching quality and promoting academic research, showing the efforts and achievements of TCM education in the academic and teaching practice aspects. The third quadrant mainly concentrates on topics related to grassroots medical services and talent cultivation. These documents emphasize the role of TCM in grassroots medical services and how to cultivate suitable talents through educational policies to meet the needs of grassroots medical services, reflecting the policy’s focus on strengthening rural medical services. The fourth quadrant gathers topics related to the specialization of TCM education and degree settings. These documents discuss the professionalization, institutionalization, and degree settings in TCM education, reflecting the strategies of TCM education in specialized and higher education development. From the figure, it can be seen that the topic documents in each quadrant have a clear trend of classification concentration, reflecting the targeted adjustments and optimizations made by TCM education policies in multiple dimensions. The first quadrant focuses on the development of the educational system and curriculum reform, the second quadrant highlights the improvement of academic research and teaching quality, the third quadrant pays attention to the strengthening of grassroots medical services and talent cultivation, and the fourth quadrant focuses on the specialization and higher education development of TCM education. This classification concentration trend further verifies the diversified promotion of TCM education policies at different levels.

**Figure 3.**
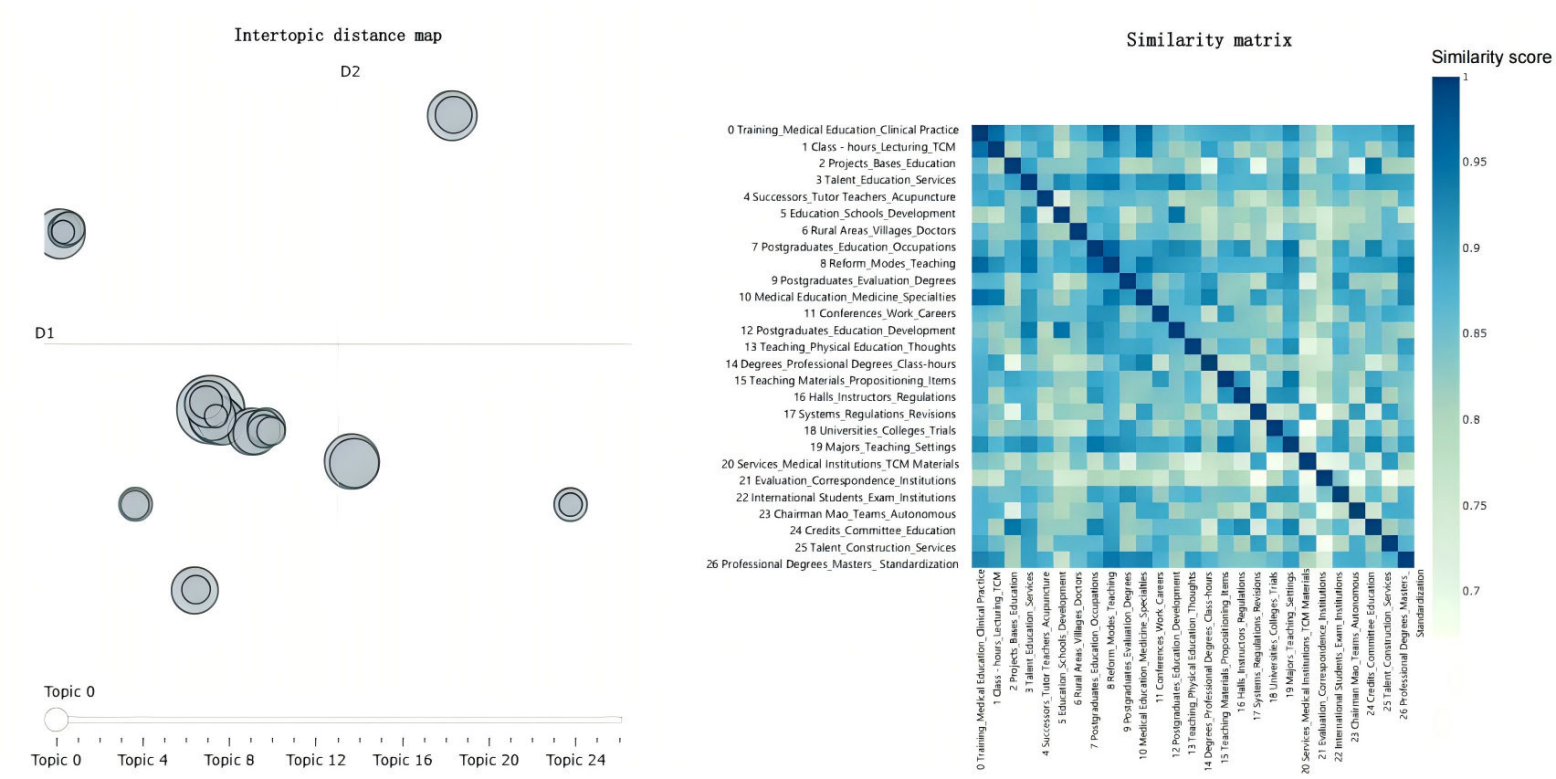
Visualization of traditional Chinese medicine education policy topic distance and cosine similarity. TCM: traditional Chinese medicine.

Based on the identified topics, the study used the model.visualize-topics() function to generate an interactive visualization of all topics, as presented in Figure 3. Each circle in the graph represents a topic, with its size indicating the frequency of the topic’s appearance in all documents. The distance between circles represents the similarity between topics, with closer distances indicating higher similarity. It can be seen that topic 0 and topic 1 are the most frequently occurring topics, involving key issues such as curriculum settings, clinical teaching, and teaching methods in TCM education. In addition, multiple topics such as topic 5, topic 7, topic 8, and topic 9 are clustered in the lower left corner of the graph, indicating higher similarity among these topics. They cover common aspects of TCM education policies, such as educational reform, improvement of teaching methods, and educational management.

**Figure 4.**
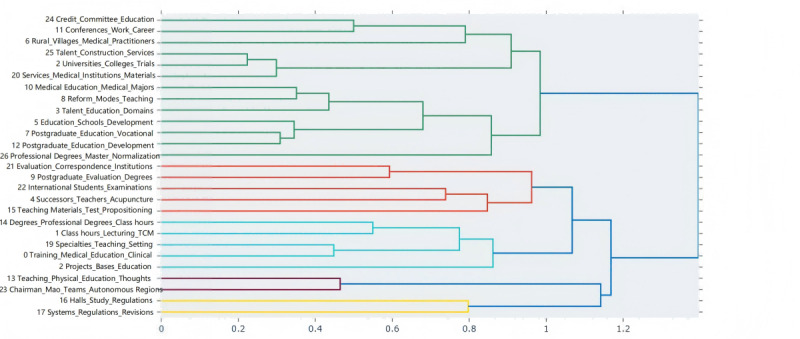
Hierarchical clustering of topics based on cosine similarity. TCM: traditional Chinese medicine.

To understand the potential hierarchical structure of the topics, hierarchical clustering was performed on the relationships between topics based on cosine similarity, and the clustering results were visualized, as shown in Figure 4. The hierarchical relationships between topics can be intuitively understood from the figure. For example, topic 18 and topic 24 are located on the same clustering branch. These topics mainly involve academic development, degree settings, and overall educational development in higher education, which are important components of the systematic construction of TCM education. Topic 21 and topic 9 are clustered together, reflecting the policy’s emphasis on standardization processes such as professional assessment, educational standards, and school management. This ensures consistency in educational content and management methods, thereby improving educational quality and professional standards. Topic 14 and topic 1 form a distinct clustering branch, indicating the policy’s focus on maintaining and developing the unique disciplinary advantages of TCM education through specialized education, curriculum design, and professional education content. Topic 13 and topic 23 are clustered together, showing a common focus on academic inheritance and team building. The policy promotes the standardized management of TCM education through these topics. Topic 16 and topic 17 are clustered together, involving topics such as schools, regulations, old medicine, and abolition, indicating the marginalization and stagnation of TCM education.

### Evolutionary Analysis of TCM Education Policy Topics

**Figure 5.**
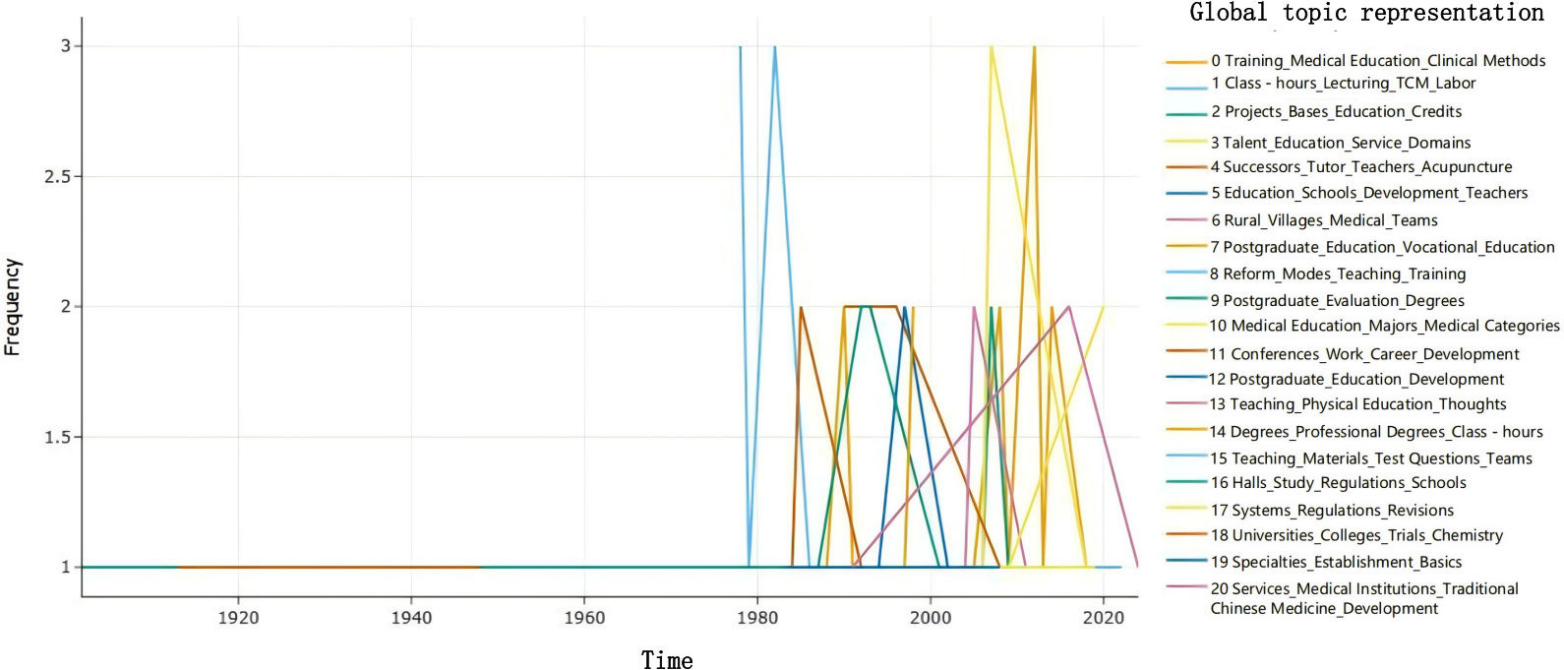
Evolutionary trends of traditional Chinese medicine education policy topics. TCM: traditional Chinese medicine.

The DTM of BERTopic can intuitively present the research hotspots and scholar attention changes in the field, facilitating the analysis of the evolutionary trends of various research directions in information resource management. Based on the model results outlined in the preceding analysis, further analysis was conducted on the historical evolution and trends of the extracted topics over time, as shown in Figure 5. It can be seen that before the 1970s, the occurrence of topics was relatively sparse, mainly focusing on basic education and training issues. After the reform and opening up, the frequency of topic occurrence increased significantly, indicating high policy activity during this period. The alternating appearance of topics suggests that TCM education policies underwent numerous reforms and adjustments during this period. For example, topic 2 and topic 10 frequently appeared during this period, indicating increasing attention to the specialized development of TCM education, such as the construction of education bases, project management, and professional education. In the 21st century, the frequency of multiple topics increased significantly and alternated frequently, indicating the complexity and diversity of policies during this period. This suggests that with the development of society, the field of TCM education is continuously adapting to new demands, and policies are constantly being adjusted and evolved accordingly. Overall, over time, the themes of TCM education policies have become richer and more complex, reflecting the developmental trajectory and evolving academic focus of TCM education policies.

## Discussion

### Evolutionary Trajectory of TCM Education Policies

Drawing on the policy cycle theory [17] and the multiple streams framework [18], this study systematically analyzes the evolutionary trends of TCM education policies across multiple key topics. These theoretical frameworks offer a structured lens to interpret how policy problems are identified, solutions formulated, and implemented over time, thereby enhancing the interpretability of the 5-stage division of TCM education policy evolution [19].

Policy cycle theory posits that policies undergo sequential stages of agenda setting, formulation, implementation, evaluation, and termination or continuation. In the context of TCM education, this theory helps understand how policy makers have navigated challenges and opportunities at each stage to advance the systematization, standardization, specialization, and normalization of TCM education [20]. The multiple streams framework, on the other hand, emphasizes the confluence of problem streams, policy streams, and political streams in driving policy change. This perspective elucidates how external factors, such as social needs, technological advancements, and political climates, have interacted to shape the evolution of TCM education policies [21]. Through the analysis of national-level policy texts using the BERTopic model, we have identified the following evolutionary stages of TCM education policies.

Through the analysis of national-level policy texts using the BERTopic model, we have identified the following evolutionary stages of TCM education policies:

#### Marginalization Stage (1840-1949)

During this period, influenced by the social and political upheaval and the influx of Western medicine, TCM education faced marginalization. The lack of a stable political environment and the dominance of Western medical concepts in the policy agenda hindered the development of TCM education, aligning with the problem stream and political stream dynamics described in the multiple streams framework.

During this period, policy themes focused on “abolishing traditional Chinese medicine” and “prohibiting old medical schools” [22]. Specifically, policymakers believed that TCM could not be verified within the framework of modern science and thus did not conform to contemporary educational principles. This perspective was reflected in several important educational policy documents, such as the “Regulations for the Establishment of Universities (Gui Mao School System)” and the “University Regulations (Ren Zi Gui Chou School System),” which explicitly called for the abolition of traditional TCM education and prohibited the establishment of any form of old medical schools [23,24]. During this time, the government’s attitude toward TCM education was extremely negative, considering it unscientific and unsystematic, and attempted to exclude it entirely from the national formal education system through coercive educational reforms. Under government suppression, TCM educational institutions were closed one after another, and the traditional TCM education model fell into a state of stagnation. This situation led to a sharp decline in the overall influence of TCM in Chinese society, and its very existence was facing unprecedented challenges. However, despite the systemic suppression of TCM education at the policy level, the demand for and support of TCM among the public did not completely disappear. Many TCM practitioners and supporters continued to preserve and develop this ancient medical system through private tutoring and family inheritance, trying to sustain TCM in a difficult environment. Although this informal education model was far smaller in scale and influence compared with the government-supported modern education system, it preserved the basic knowledge and skills of TCM to a certain extent, providing an important foundation for the later revival of TCM education.

#### Normalization Stage (1949-1978)

After the founding of the People’s Republic of China, the government began to recognize the value of TCM, and policies were formulated to normalize TCM education. This stage reflects the agenda-setting and formulation phases of the policy cycle, as policy makers worked to integrate TCM into the national education system.

The policy themes during this period mainly focused on “restoring TCM education,” “Western medicine practitioners learning TCM,” “master-apprentice TCM education,” and “inheriting the academic experience of senior TCM practitioners” [25]. With the establishment of the new China, the national ideology reevaluated the value of TCM and began to protect and develop it as an important part of Chinese culture. Against this backdrop, the government issued a series of policies aimed at restoring and standardizing TCM education. For example, the “Notice on the Regulations for the Organization of TCM Advanced Study Schools and Classes” and the “Instruction on Carrying Out Master-Apprentice TCM Education” clarified the measures for the restoration of TCM education and attempted to combine modern education with traditional master-apprentice inheritance to ensure the effective transmission and development of TCM knowledge and skills. The policy of “Western medicine practitioners learning TCM” was a significant orientation during this period. The government hoped to integrate the strengths of both traditional and modern medicine by encouraging Western medical practitioners to study TCM, thereby addressing the shortage of medical resources at the time. Additionally, the government advocated for the continuation of traditional TCM knowledge through the “master-apprentice” approach, providing a dual safeguard for the revival of TCM education [26]. Furthermore, recognizing that the essence of TCM was often preserved in the experience of senior practitioners, the government issued the “Urgent Notice on Inheriting the Academic Experience of Senior TCM Practitioners” to ensure that this valuable knowledge would not be lost over time. This policy demonstrated the government’s emphasis on balancing inheritance and innovation in TCM education and its commitment to preserving and promoting the core aspects of TCM through institutional means [27]. During this stage, the normalization of TCM education was reflected not only in policy making but also in the gradual improvement of the educational system. The government established TCM colleges nationwide and formulated unified teaching syllabuses and textbooks, such as the “Opinions on the Compilation of Teaching Syllabuses and Textbooks for TCM College Courses.” These measures ensured the standardization of TCM education, leading to its expansion in scale and enhancement in content and quality.

#### Specialization Stage (1978-1999)

With the reform and opening-up policy, TCM education entered a stage of specialization. The emphasis on developing specialized TCM education programs and improving the quality of TCM education can be seen as a response to the evolving social needs and technological progress, in line with the implementation and evaluation stages of the policy cycle.

After China implemented the reform and opening-up policy in 1978, TCM education entered a new stage characterized by specialization. The policy themes during this period mainly focused on “promoting and developing TCM education,” “seven-year programs,” “teaching content,” and “academic inheritance.” These themes not only reflected the government’s attention and investment in TCM education but also demonstrated the deepening and expansion of TCM education within the modern educational system. “Promoting and developing TCM education” was the core policy orientation of this stage [28]. The government recognized the unique value of TCM education in the modern medical system and issued a series of policies aimed at supporting and developing TCM education. For example, the “Report on Seriously Implementing the Party’s TCM Policy and Solving the Problem of Succession in the TCM Workforce” emphasized the issue of TCM talent cultivation and proposed strengthening education to address the shortage of successors in the TCM field [29]. The “seven-year program” was a significant innovation in TCM education during this period. To improve the quality of TCM education, the government began to extend the duration of TCM education from the traditional 5-year program to a 7-year program. This measure not only increased the students’ learning time but also broadened and deepened the educational content, enabling students to graduate with more solid theoretical knowledge and practical skills in TCM. The “Notice on the Trial Implementation of Seven-Year Higher TCM Education” officially launched this educational reform, marking a step toward higher academic standards in TCM education. In terms of teaching content and academic inheritance, the government ensured the integrity and systematic nature of TCM education through a series of policies. For example, the “Opinions on Strengthening Clinical Teaching in Higher TCM Education” emphasized the importance of clinical teaching, ensuring that students gained sufficient clinical practice experience in addition to theoretical learning. This combination of theory and practice made TCM education more practical and capable of cultivating TCM talents that met societal needs. Moreover, the government focused on the academic inheritance of TCM education, ensuring the intergenerational transmission of traditional TCM knowledge and skills [30]. The “1988‐2000 TCM Education Development Strategy Plan” set clear academic inheritance goals, using apprenticeship and other inheritance methods to ensure that the core knowledge of TCM was not diluted by the modernization process.

#### Systematization Stage (1999-2012)

Entering the 21st century, TCM education entered a new stage of systematization. During this stage, policies aimed at systematizing TCM education, integrating various aspects of education, including curriculum design, teaching methods, and teacher training. This comprehensive approach to policy development is consistent with the continuous improvement and refinement process within the policy cycle.

The policy themes during this period mainly focused on “continuing education in TCM,” “reform of management systems and mechanisms,” and “internationalization of TCM education.” These themes reflected the government’s intention to improve the overall level and international competitiveness of TCM education through the perfection of the educational system, deepening of management system reforms, and promotion of international cooperation [31]. “Continuing education in TCM” was a key focus of policies during this period. With the development of society and the changing medical demands, the government realized that traditional TCM education alone was insufficient to meet the complex needs of modern medicine. Therefore, continuing education became an important component of TCM education. For example, the “TCM Continuing Education Fifteenth Five-Year Plan” and the “Regulations on TCM Continuing Education” clarified the goals and implementation methods of TCM continuing education, requiring the enhancement of TCM practitioners’ knowledge and skills through continuing education to address new medical challenges. “Reform of management systems and mechanisms” was another important theme in the development of TCM education during this stage [32]. As educational system reforms deepened, the management and operation mechanisms of TCM education also underwent significant adjustments. For example, the “Opinions on Several Issues Concerning TCM Education” and the “TCM Innovation Development Plan Outline (2006‐2020)” proposed the direction of management system reform in TCM education, aiming to improve the efficiency and quality of TCM education through educational system and mechanism reforms. “Internationalization of TCM education” was also an important policy orientation during this stage. With the enhancement of China’s international status and the growing global influence of TCM, the government began to actively promote the internationalization of TCM education [33]. Policy documents such as the “Several Opinions of the State Council on Supporting and Promoting the Development of the TCM Cause” and the “Basic Requirements for the Establishment of TCM Undergraduate Education in Colleges and Universities (Trial)” clarified the goals and strategies for the internationalization of TCM education, encouraging TCM colleges and universities to cooperate with international academic institutions to promote the globalization of TCM education and research.

#### Standardization Stage (2012 to Present)

In recent years, TCM education policies have focused on standardization, aiming to establish unified standards for TCM education at home and abroad. This stage reflects the efforts to enhance the international competitiveness of TCM education and ensure its quality and consistency, in line with the ongoing evaluation and potential continuation phases of the policy cycle.

Since 2012, TCM education in China has entered a new stage of standardization. The policy themes during this period mainly focused on “standardization,” “integration of medical and educational collaboration,” “cultivation of general practitioners,” and “high-quality development.” These themes reflected the government’s intention to ensure the high quality and level of TCM education through standardization, further enhancing its international competitiveness. “Standardization” has been a core theme of TCM education policies during this stage. To achieve the normalization and scientification of TCM education, the government has issued a series of policy documents specifying the standardization requirements for TCM education [34]. For example, the “Law of the People’s Republic of China on Traditional Chinese Medicine” and the “Several Opinions on Further Promoting the ‘5+3’ Integrated Medical Talent Training Work” detailed the teaching standards, curriculum settings, and talent training models for TCM education. “Integration of medical and educational collaboration” has been an important development model for TCM education during this stage. The government realized that further development of TCM education required a closer combination with medical practice. Therefore, the “Guiding Opinions on Deepening the Reform and Development of TCM Education through Medical and Educational Collaboration” was issued, specifying the requirements for medical and educational collaboration and emphasizing the improvement of TCM students’ practical abilities and comprehensive quality through the integration of teaching and clinical practice. “Cultivation of general practitioners” has been another important theme in TCM education policies during this stage. With the increasing demand for general practitioners in society, the government began to introduce a general practitioner training model into TCM education. For example, the “Implementation Measures for the Standardized Training of TCM Resident Physicians (Trial)” and the “Guiding Opinions on Deepening the Succession Education of TCM” proposed a combination of standardized training and master-apprentice education to cultivate general TCM practitioners with comprehensive capabilities. “High-quality development” has been the overall goal of TCM education during this stage. Against the backdrop of the rapid development of the global health sector, the government required TCM education to focus on quality and promote high-level, high-standard development [35]. For example, the “Notice on the Implementation of the ‘Double Ten Thousand Plan’ for First-Class Undergraduate Major Construction” and the “Several Policy Measures for Accelerating the Characteristic Development of TCM” emphasized the improvement of teaching quality, optimization of curriculum settings, and strengthening of scientific research innovation to achieve high-quality development in TCM education.

### Logic Behind the Evolution of TCM Education Policies

#### Logic of Balancing Modernization and Traditional Inheritance

The evolution of TCM education policies in China centers on the balance between modernization and traditional inheritance [36]. Since the mid-19th century, the invasion of Western powers and the introduction of modern science had a profound impact on traditional Chinese culture. In an effort to adapt to the new external environment, policymakers introduced the Western medical education system and attempted to abolish traditional TCM education to promote the modernization of Chinese medicine. This reflected the then-prevailing belief that modernization was the only path to national strength, and traditional TCM education was seen as an obstacle to this process. However, this logic of excluding tradition did not fully succeed. Despite the policy attempts to suppress TCM education, TCM, as an important part of Chinese culture, did not disappear due to policy pressure. Many TCM practitioners continued to preserve and spread TCM knowledge through private tutoring and apprenticeship, laying the foundation for the survival of TCM [37]. After the founding of the People’s Republic of China in 1949, the state reevaluated the value of TCM and began to restore and standardize TCM education. For example, the “Notice on the Regulations for the Organization of TCM Advanced Study Schools and Classes” was a typical example, reflecting the state’s emphasis on TCM education and aiming to restore the legitimate status of TCM in the medical system through the reconstruction of the educational system. This logic reflects the state’s gradual recognition in the modernization process that Western medicine alone could not meet China’s needs, and a balance between modernization and traditional inheritance had to be found [38].

#### Logic Driven by Political and Social Demands

The evolution of TCM education policies in China has been deeply influenced by both political environment and social demands, with each stage of policy change reflecting the political background and social needs of the time. After the founding of New China, the government rerecognized the importance of TCM as an important part of Chinese traditional culture, and policies such as the “Instruction on Carrying Out Master-Apprentice TCM Education” were issued in the 1950s to restore the legitimate status of TCM education and address the challenge of medical resource shortages. During the reform and opening-up period, with rapid economic development and changes in social structure, TCM education policies began to emphasize specialized development to meet the diverse medical service needs. The “1988‐2000 TCM Education Development Strategy Plan” was a representative policy of this period, proposing to extend the academic system, enrich the curriculum content, and strengthen clinical practice to cultivate high-quality talents with TCM characteristics. Entering the 21st century, with the enhancement of China’s international status and the acceleration of globalization, the international demand for TCM increased, and the government integrated international considerations into educational policies. For example, the “Several Opinions of the State Council on Supporting and Promoting the Development of the TCM Cause” explicitly proposed to promote the internationalization of TCM education, encouraging TCM colleges and universities to cooperate with international academic institutions to enhance its global influence. These policies not only responded to domestic and international demands for TCM but also reflected the strategic goal of enhancing China’s cultural soft power through the internationalization of TCM education.

#### Logic of Adaptation to Reform and Innovation

Reform and innovation are key logics in the evolution of TCM education policies in China. With social development and technological progress, TCM education faces new challenges and opportunities, and policymakers have gradually placed reform and innovation at the core of policymaking. Since the reform and opening-up, Chinese society has undergone tremendous changes, and TCM education policies have also entered a new stage oriented by innovation. In the mid-1980s, the government realized that merely restoring and standardizing TCM education was insufficient to meet modern demands, and it was necessary to enhance the quality and international competitiveness of education through reform and innovation. For example, the “Report on Seriously Implementing the Party’s TCM Policy and Solving the Problem of Succession in the TCM Workforce” emphasized educational model innovation through academic system reform, curriculum enrichment, and teaching method improvement. The introduction of the 7-year program was a specific manifestation of this innovative logic [39], and the “Notice on the Trial Implementation of Seven-Year Higher TCM Education” marked a milestone in the modernization of TCM education. With the upgrading of medical service demands, standardization became a focus of reform in the early 21st century. The government implemented standardization policies such as the “Law of the People’s Republic of China on Traditional Chinese Medicine” and the “Several Policy Measures for Accelerating the Characteristic Development of TCM” to ensure the consistency and global competitiveness of educational quality. This standardization not only guaranteed high-quality output but also made TCM education more acceptable and recognizable by the international community.

#### Logic of Integration of Cultural Confidence and National Strategy

The deep logic of TCM education policies includes the integration of cultural confidence and national strategy. As an important part of Chinese traditional culture, TCM education policies are not only related to medical education but also bear the mission of cultural inheritance and national strategy. In the early days of New China, the restoration of cultural confidence was a key driving force for the revival of TCM education policies. With the enhancement of China’s comprehensive national power, the government has gradually strengthened its emphasis on TCM education, consolidating its important position in national culture and social development through institutional means. For example, the “Urgent Notice on Inheriting the Academic Experience of Senior TCM Practitioners” emphasized the protection and inheritance of the valuable experience of senior TCM practitioners, laying the foundation for the long-term development of TCM. At the national strategy level, the evolution of TCM education policies has always been closely linked to the overall national development strategy. After the reform and opening-up, with the enhancement of China’s economy and international influence, the government has paid more attention to the globalization and standardization of TCM education. This not only reflects cultural confidence but also demonstrates the important role of TCM education in showcasing China’s soft power [40]. The integration of cultural confidence and national strategy runs through all stages of TCM education policy evolution, promoting the modernization and internationalization of TCM education and enhancing the global influence of Chinese culture.

#### Policy Effectiveness in TCM Education

Assessing policy effectiveness is crucial as it reveals the practical impact of policy initiatives and provides insights for future improvements, aligning with the tenets of policy evaluation research. During the Standardization Stage (2012 to Present), TCM education policies aimed to establish unified standards both domestically and internationally. A notable case is the implementation of the “Undergraduate Medical Education Standards-Traditional Chinese Medicine Major (Provisional) (2012)” in China. This policy stipulated specific requirements for curriculum design, teaching resources, and faculty qualifications, leading to a significant improvement in the quality of TCM education. For instance, Henan University of Chinese Medicine achieved a 99.33% course evaluation coverage rate in 2018‐2019, while Zhejiang University of Chinese Medicine reported an 89.22% national licensing examination pass rate in 2020, 24.6 percentage points above the national average [41].

In the Specialization Stage (1978‐1999), policies promoting the development of specialized TCM education programs also demonstrated remarkable effectiveness. For example, the establishment of TCM clinical specialization courses in major TCM universities across China was a direct result of policy support. These courses focused on in-depth training in areas such as acupuncture, Chinese herbal medicine, and TCM diagnosis. Take Beijing University of Chinese Medicine as a case. Beijing University of Chinese Medicine enrolled its first group of 36 master’s students in 1978, and clinical program enrollment increased by 40% from 1985 to 1995 [42]. Moreover, 65.17% of Chengdu University of Traditional Chinese Medicine graduates serve at the grassroots level, with more than 80% relevance to their major [43]. This case study illustrates how specialization-oriented policies successfully cultivated high-quality TCM professionals, meeting the practical needs of the industry.

However, policy effectiveness was not uniformly positive across all stages. During the Marginalization Stage (1840‐1949), due to social unrest and the influence of Western medicine, TCM education policies struggled to achieve their intended goals. The lack of stable political support and limited resources meant that TCM education institutions faced difficulties in maintaining educational quality and scale. For instance, many traditional TCM private schools were forced to close, and the number of TCM students declined sharply during this period [44]. This historical case highlights the importance of a conducive social and political environment for policy implementation and effectiveness.

In conclusion, by analyzing these case studies, it is evident that TCM education policies have achieved varying degrees of success. Effective policies often require a combination of clear goals, adequate resource allocation, and a supportive social context. Insights from these case studies can inform future policy making, helping to optimize TCM education development and better meet the needs of society and the medical field.

#### Future Trends of TCM Education Policies

First, the deepening of standardization and internationalization. In the future, TCM education needs to further promote standardization, especially in the context of globalization, where alignment with international standards will become crucial. With China’s increasing influence in the international community, TCM, as a representative of Chinese culture, will face more international cooperation demands in its educational system. Accelerating the formulation and promotion of TCM education standards and incorporating TCM courses into the international medical education system will be key to promoting its global dissemination, attracting more international students and researchers, and enhancing the international influence of TCM, thereby promoting the diversification and innovation of education.

Second, the strengthening of innovation and integration with technology. TCM education needs to place greater emphasis on the integration with modern technology, with information technology and biotechnology becoming important drivers. The development of artificial intelligence, big data, blockchain, and other technologies will greatly improve the efficiency and quality of TCM education. Digital platforms and web-based education tools may be more widely applied in TCM education, providing convenient ways to access knowledge and virtual laboratories to enhance students’ learning outcomes and practical abilities.

Third, the balanced development of cultural confidence and globalization. With the enhancement of China’s international status, TCM education needs to maintain cultural confidence in the context of globalization. This confidence should be reflected not only in the inheritance and innovation of TCM educational content but also in how to play a greater role in global health governance. Future policies will place greater emphasis on the cultural export of TCM, showcasing its unique value through international exchanges, academic cooperation, and cultural activities to enhance the international image of TCM and China’s discourse power in international medical education.

Fourth, the expansion of policy support and diversified cooperation. Future TCM education policies should place greater emphasis on policy support and diversified cooperation. Through measures such as financial investment, legal protection, and talent introduction, support should be provided for TCM colleges and universities in their exploration of scientific innovation, international cooperation, and digital transformation. At the same time, cooperation with international organizations, multinational corporations, and nongovernmental organizations should be strengthened to promote the global allocation and sharing of TCM educational resources. This diversified cooperation will attract more global resources for TCM education, promoting its continuous innovation and development.

### Conclusions and Outlook

The evolution of TCM education policies is not only the result of historical development and social demands but also a comprehensive reflection of cultural inheritance, innovative reform, and international strategy in China’s modernization process. From the early marginalization to the normalization after the founding of New China, from the specialized development during the reform and opening-up period to the standardization and internationalization in the 21st century, each adjustment and evolution of TCM education policies reflects China’s rethinking and redefinition of the positioning of TCM education in different historical stages. The core logic of this policy evolution process not only provides an effective framework for understanding the past of TCM education but also offers important clues for predicting its future development direction. In the future, the formulation of TCM education policies should maintain the traditional advantages of TCM while actively addressing new challenges brought by modern technology and globalization, promoting the continuous optimization and improvement of the TCM education system. It is necessary not only to meet the domestic demand for medical services but also to inherit and promote Chinese culture globally, becoming an important bridge connecting Eastern and Western medicine. Through continuous policy innovation and improvement, TCM education will contribute more to the development of health care in China and around the world.

## Abbreviations

c-TF-IDF: class-based Term Frequency–Inverse Document Frequency
DBSCAN: Density-Based Spatial Clustering of Applications with Noise
DTM: dynamic topic modeling
HDBSCAN: Hierarchical Density-Based Spatial Clustering of Applications with Noise
LDA: Latent Dirichlet Allocation
TCM: Traditional Chinese medicine
UMAP: Uniform Manifold Approximation and Projection
